# NETs Lead to Sympathetic Hyperactivity After Traumatic Brain Injury Through the LL37-Hippo/MST1 Pathway

**DOI:** 10.3389/fnins.2021.621477

**Published:** 2021-04-29

**Authors:** Kaixin Zhu, Yibai Zhu, Xiaoxiang Hou, Wen Chen, Xiaolin Qu, Yelei Zhang, Zhenxing Li, Chunhui Wang, Jigang Chen, Liquan Lv, Junyu Wang, Danfeng Zhang, Lijun Hou

**Affiliations:** Department of Neurosurgery, Changzheng Hospital, Naval Medical University, Shanghai, China

**Keywords:** traumatic brain injury, sympathetic hyperactivity, NETs, LL37-Hippo/MST1, pathogenesis

## Abstract

**Background:** Paroxysmal sympathetic hyperactivity (PSH) is one of the important reasons for the high mortality and morbidity of traumatic brain injury (TBI). We aim to explore the role of the neutrophil extracellular traps (NETs) in the pathogenesis of sympathetic hyperexcitability after TBI and the underlying mechanisms, providing evidence for clinical treatment.

**Methods:** Enzyme-linked immunosorbent assay was used to assess the plasma metanephrine and normetanephrine levels which represented the variation of the sympathetic system after TBI with rat diffuse axonal injury (DAI) model. NETs in the paraventricular nucleus (PVN) and circulating blood were examined using immunofluorescence and flow cytometry. Neutrophils-microglia co-culture system was established to further explore the effect of NETs on PSH and its mechanisms.

**Results:** After TBI, metanephrine and normetanephrine levels began to increase at 9 h and peaked at 72 h. After the injury, the level of NETs kept increasing at 24 and 72 h in the PVN. A positive correlation was found between the concentration of the PVN NETs and blood catecholamine. Flow cytometry of peripheral blood cells revealed that NETs level in the injury group was higher than that in the control group. Immunofluorescence results confirmed the presence of NETs in the PVN after TBI. The positive result of immunoprecipitation suggested a correlation effect between LL37 and P2 × 7. Peptidyl arginine deiminase-4 (PAD4) inhibitor could inhibit the expression levels of MST1, YAP, and IL-1β. The hippo/MST1 pathway inhibitor could inhibit the expression levels of YAP and IL-1β.

**Conclusion:** NETs formation in the PVN might be associated with sympathetic hyperactivity after TBI, which might relate to the activation of microglia cells and increased secretion of IL-1β via the hippo/MST1 pathway.

## Introduction

Globally, there are more than 60 million new cases of traumatic brain injury (TBI) each year, and the incidence rate is still increasing year over year (Dewan et al., 2018). It is estimated that nearly 50% of the world’s population are affected by TBI in their lifetime (Maas et al., 2017). Of all site-specific trauma, TBI is the most frequent cause of death and disability (Rubiano et al., 2015). Paroxysmal sympathetic hyperactivity (PSH) is often secondary to TBI, accounting for about 8–33% of TBI patients (Bueno González et al., 2014). It is mainly manifested as paroxysmal hyperexcitability of the sympathetic nervous system and abnormal motor system and its clinical symptoms include tachycardia, tachypnea, diaphoresis, fever, hypertension, and dystonic posturing (Hasen et al., 2019). PSH is one of the main reasons for the high mortality and morbidity of TBI (Meyfroidt et al., 2017).

The pathological hypotheses of secondary sympathetic excitation after TBI abound. One of them holds that the pathological basis of sympathetic excitation after TBI is the interruption of the anterior descending spinal cord pathway (cortical inhibition center – hypothalamus – diencephalon – brainstem) after injury, which innervates sympathetic activity. As a result, the normally inhibited spinal cord excitation loop was disinhibited and the output of sympathetic nerve activity increased (Meyfroidt et al., 2017). Previous studies have found that the PVN is a key region that regulates sympathetic excitation in the central nervous system in various pathological conditions, such as hypertension, myocardial infarction, heart failure, and so on (Meyfroidt et al., 2017; Koba et al., 2018).

Recent studies have shown that accumulated neutrophils can release neutrophil extracellular traps (NETs) which are composed of enucleated chromatin and granular substances (Papayannopoulos, 2018). NETs are involved in a number of pathophysiological processes including tumor cell metastasis, inflammation, ischemia-reperfusion injury, autoimmunity, and so on (Pietronigro et al., 2017). Although the mechanism of NETs formation (NETosis) is not yet fully clear, a growing number of studies suggest that NETs play an important role in the development of Alzheimer’s disease, ischemic brain injury, and many other central nervous systems diseases. In pathological conditions, neutrophils in cerebral micro vessels and parenchyma form NETs actively, promoting inflammatory response, aggravating cerebrovascular and parenchymal damage (Pietronigro et al., 2017; Valles et al., 2017). Central nervous system inflammation is the important pathological basis of sympathetic excitation in hypertension, myocardial infarction and other pathological conditions (Wang et al., 2019). Moreover, we have previously found that reactive oxygen species in the rostral ventrolateral medulla account partially for the PSH after TBI, which is the classical way of NETosis. Therefore, it’s worth exploring the role of NETs in PSH after TBI.

The main components of NETs include neutrophil elastase, LL37, histone Citrullinated histone H3 (CitH3), Cathepsin G, MMP9, myeloperoxidase (MPO), and depolymerized DNA (Albrengues et al., 2018). Among them, LL37 is an agonist of the P2 × 7 receptor, which is a trimer ATP-gated cationic channel and highly expressed on the cell membrane of microglia and monocytes. P2 × 7 can promote the activation and proliferation of microglia in TBI, ischemic brain injury, and Alzheimer’s disease (Monif et al., 2010). Elssner et al. (2004) found that LL37 could promote the secretion of IL-1β in monocytes through the P2 × 7 receptor. As one of the important inflammatory mediators after TBI, IL-1β can participate in sympathetic excitation by regulating levels of excitatory/inhibitory neurotransmitter and neuronal activity (Wang et al., 2019; Yan et al., 2019). The increased level of IL-1β in the PVN is proved to participate in the sympathetic excitation in hypertension, myocardial infarction, and heart failure (Liu et al., 2014; Qi et al., 2016; Wang et al., 2019). We assume that LL37 in NETs regulates the secretion of IL-1β, which might be related to the activation of microglia and PSH after TBI. Therefore, the purpose of this article is to find out the pathogenesis of sympathetic hyperexcitability after TBI, providing evidence for clinical management.

## Materials and Methods

### Animals

Sprague Dawley (SD) rats, male, weighing 230–280 g, were selected and provided by the Animal Experiment Center of the Second Military Medical University. Feeding and experimental procedure of experimental animals were in accordance with the Guide for the Care and Use of Laboratory Animals published by the US National Institutes of Health (NIH publication No. 85–23, revised 1996). It was supervised by the Animal Care and Use Committee of the Second Military Medical University. During the process, efforts were made to minimize the pain and number of animals used.

### Experimental Design

#### To Assess the Effects of TBI on the Sympathetic System

Seventy rats were randomly assigned to either DAI (*n* = 40) group or the sham-injury (*n* = 30) group. Animals in the DAI group and the control group, respectively, were divided into five subgroups (9, 24, 48, 72, and 96 h, *n* ≥ 6). At five time points (9, 24, 48, 72, and 96 h) after the DAI attack, blood was taken from the heart of the rats after anesthesia, then the rats were perfused with normal saline. After perfusion, rats were beheaded and their brains were harvested. Blood samples were collected in ethylenediamine tetra acetic acid (EDTA) tubes for the determination of plasma catecholamine, and brain tissue samples were immersed in liquid nitrogen and stored in −80°C refrigerator for subsequent experiments. CitH3, a marker of NETs, in the PVN of rats at different time points was detected using western blot (WB).

#### In Order to Examine the Existence of NETs in the PVN After TBI

Sixteen male SD rats were randomly divided into the DAI group (*n* = 10) and the control group (*n* = 6). Blood and brain collection, as well as the perfusion process, were conducted as mentioned above at 72 h after DAI. Blood samples were collected into an EDTA tube for cell flow cytometry to detect the formation of NETs. Brain tissue samples were kept in paraformaldehyde for immunofluorescence detection of NETs in the PVN.

#### In Order to Further Explore the Effect of NETs on PSH

Neutrophils were isolated from circulating blood at 72 h after DAI. After 2 days of culture, 10^7^ microglia (HAPI, FH-H262) and 10^7^ rat neutrophils were taken together in one well of a 6-well plate and co-cultured for 24 h. They were divided into four groups: DAI group, DAI, and YW3-56 (5 μM, PAD4 inhibitor which could inhibit the formation of NETs) group, DAI and XMU-MP-1 (50 nM, Hippo pathway inhibitor) group, control group. After 24 h of culture, the interaction between LL37 and P2 × 7 was examined, and the levels of MST1, YAP, and IL-1β were measured.

### DAI and Sham-Injury

Diffuse axonal injury and sham-injury were conducted according to a standard protocol as previously described (Chen et al., 2019). Briefly, rats were anesthetized with isoflurane, then the head was fixed on the DAI injury device. The head was rotated about 75° (4.68 ms, 1.6 × 1.815 rad/s) on the coronal plane, and moved 1.57 cm (4.66 ms, 3.4 × 102 cm/s) horizontally to realize the angular/linear acceleration and deceleration injury of the head. Then the tongue of the rat was pulled out with ophthalmic forceps to prevent it from suffocating. Rats in the control group received anesthesia and were fixed on the same device without a strike.

### Blood Sample and the PVN Tissue Collection

After anesthesia, blood and brain tissue samples were collected as mentioned above and stored for subsequent experiments. The preserved brain tissue was put into the cryosection machine at −20°C for 30 min to prevent nuclear fragmentation and bonded to the base of the cryosection machine. Knife distance was set to 50 μM. According to the brain atlas of rats, the frontal lobe was cut until stria alba disappeared, and then 24 knives (about 1,200 μM from stria alba to the PVN) were cut to obtain the anterior section containing the PVN tissue. The thick sections containing the PVN were obtained. To extract the PVN tissue, we use a fine needle to prick the edge of the PVN on the thick section according to the atlas, and then cut it out with a blade.

### Cell Culture

Rat microglia (HAPI, FH-H262) were selected. HAPI cells were adherent and rapidly growing cell lines. They were cultured in DMEM medium supplemented with 10% FBS and 1% double antibody. Cells were subcultured every 2 days, digested with 0.25% trypsin, and then 20% of them were preserved.

### Immunofluorescence

Coronal sections of brain tissue were fixed with 4% formaldehyde solution for 15 min. The sections were infiltrated with a blocking solution and sealed for 1 h at room temperature. Then anti-CitH3 (1:100, Abcam, rabbit polyclonal, Cambridge, United Kingdom) was used as primary antibody overnight at 4°C, followed by a 2 h incubation with secondary antibodies: donkey anti-rabbit-FITC (1:250, Beyotime, Shanghai, China). After washing with PBS-T for 5 min and three times, DAPI (Beyotime, Shanghai, China) was added and incubated in dark for 2 min. Then DAPI was washed out with PBS for 1 min and three times. PBS outside the specimen was wiped off with filter paper. Sections were sealed with glycerin and observed immediately under the fluorescence microscope. The sealing solution was applied overnight at room temperature. CitH3 and DAPI expression sites and their fluorescence intensity were analyzed.

### Enzyme-Linked Immunosorbent Assay

Blood samples were obtained from the heart using EDTA tubes. Following centrifugation at 3,000 rpm at 4°C, plasma samples were collected and stored at −80°C. Plasma metanephrine and normetanephrine levels were measured using rat metanephrine Elisa kit (#9070-B, Jingmei, Jiangsu, China) and normetanephrine Elisa kit (#9067-B, Jingmei, Jiangsu, China).

### qPCR

Total RNA from the rat PVN was extracted out and reverse-transcripted into cDNA (AT341, TransGen, Beijing, China). Primer synthesis: MST1-F: 5′-GCTGCGGCATCAAATCA-3′; MST1-R 5′-TGGAAAGGGTGCGAGTG-3′; YAP-F: 5′-TCGTCATGGGTCTAGTTGG-3′; YAP-R: 5′-GATGTGGC GGAGTTTCAG-3′; Actin-F: 5′-TCAGGTCATCACTAT CGGCAAT-3′; Actin-R: 5′-AAAGAAAGGGTGTAAAACGCA-3′. Then real-time quantitative PCR was performed (SG Fast qPCR Master Mix B639273, Sangon, Shanghai, China).

### WB

The expression of CitH3 in the PVN was detected by WB. In brief, the protein concentration was measured with the BCA kit (E112-01, Vazyme, Nanjing, China). The protein samples were applied to a 10% SDS-PAGE gel, followed by transferring to PVDF membrane. The membrane was blocked and incubated with primary antibody against CitH3 (Abcam, United States) overnight at 4°C. Then the membrane was incubated with secondary antibodies (1:5000) for 2 h at room temperature. Finally, the protein bands were visually detected and analyzed. Tubulin served as a loading control.

### Flow Cytometry

Flow cytometry was carried out as published before (Mozaffari Nejad et al., 2020). Briefly, cells were suspended and fixed in 4% formaldehyde for 15 min and then permeabilized using 0.2% Triton X-100 for 10 min at room temperature. Subsequently, cells were labeled with the primary antibody against CitH3 (Abcam, United States) and MPO (Proteintech, United States) which were diluted in PBS containing 1% BSA for 30 min. Cells were then washed with PBS. FITC-conjugated secondary antibodies (P0013B and A0453, Beyotime, Shanghai, China) were added and incubated at room temperature for 30 min. The stained cells were analyzed using flow cytometry (BD FACS-Aria, United States). Each experiment was assayed three times.

### Statistical Analysis

GraphPad Prism 8.0 (GraphPad Software, San Diego, CA, United States) was used to sort out and analyze the experimental data. The experimental data were expressed as mean ± standard deviation (x ± s). Comparison among groups was made using the independent sample *t*-test. A *P*-value < 0.05 was considered statistically significant.

## Results

### The Level of Sympathetic Excitation Peaked at 72 h After TBI

A total of 40 SD rats were subjected to DAI. Among them, three rats died directly after the strike, two rats died within 2 h after injury, no rat died thereafter. The survival rate was 87.5% (35/40). The main cause of death was suffocation caused by nasal bleeding. There was no death in the control group. Thirty-five surviving rats were randomly divided into five subgroups.

**FIGURE 1:**
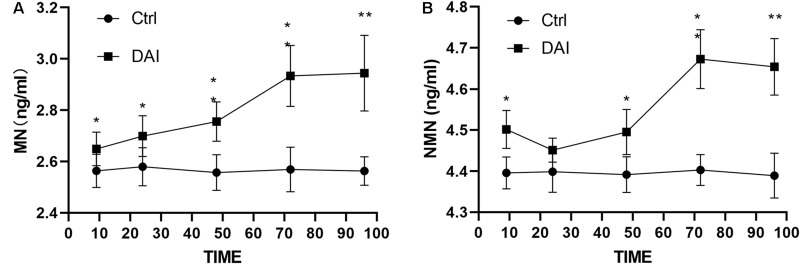
**(A)** Plasma metanephrine level in rats increased slightly at 9 h after DAI, increased significantly at 24 h (*P* < 0.05), peaked at 72 h, and then was stabilized at 96 h, but it was still higher than that in the control group (*P* < 0.01). **(B)** Plasma normetanephrine level began to increase at 9 h after DAI (*P* < 0.05), decreased slightly at 24 h, then increased again at 48 h, peaked at 72 h, and then decreased slowly at 96 h, but it was still higher than that of the control group (*P* < 0.01). Grouping: DAI group (*n* = 40) and sham-injury group (*n* = 30) (*P < 0.05 and **P < 0.01).

After DAI, an overall upward trend was found for the plasma metanephrine level, which rose from 9 h, peaked at 72 h, and tended to be stable at 96 h. It continued to be higher than that of the control group (Figure 1A). The level of plasma normetanephrine increased at 9 h, decreased slightly at 24 h, then increased again at 48 h, reached the peak at 72 h. It showed a stable trend at 96 h and continued to be higher than that of the control group (Figure 1B).

### NETs Were Activated After TBI and Reached a Relatively High Level at 72 h After TBI

**FIGURE 2:**
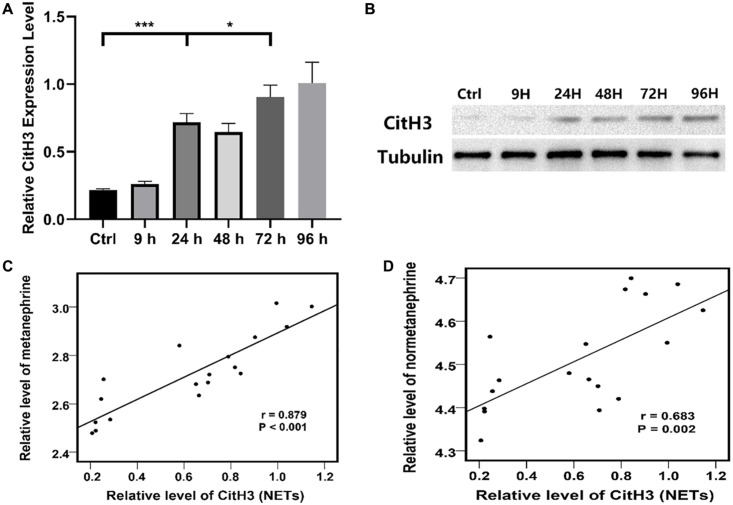
**(A)** Relative Citrullinated histone H3 (CitH3) protein expression level in the PVN of the control group and each experimental subgroup (*P < 0.05 and ***P < 0.001). **(B)** CitH3 protein expression in the PVN of the control group and each experimental subgroup were detected by WB. **(C)** Relative level of metanephrine was positive correlate to that of CitH3 (NETs) according to Pearson correlational analysis, *r* = 0.879, *P* < 0.001. **(D)** Relative level of normetanephrine was positive correlate to that of CitH3 (NETs) according to Pearson correlational analysis, *r* = 0.683, *P* = 0.002. Grouping: DAI group (*n* = 18) and sham-injury group (*n* = 18).

The expression of CitH3 in the PVN of rats was detected by WB. The results showed that CitH3 appeared in the PVN about 24 h after DAI injury, and the level of CitH3 kept increasing at 72 h with a subsequent upward trend (Figures 2A,B).

A positive correlation was found between the concentration of the PVN NETs (CitH3) and blood catecholamine (metanephrine and normetanephrine) concentrations across time points according to Pearson correlational analysis (Figures 2C,D).

**FIGURE 3:**
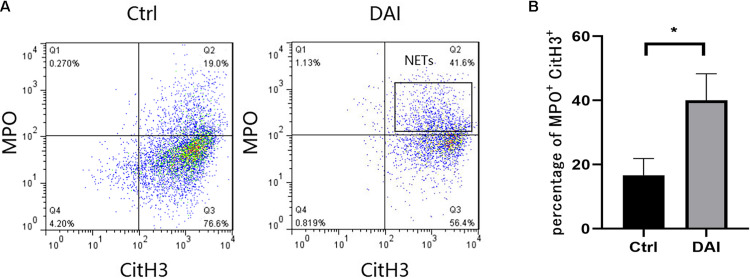
**(A)** Flow cytometric detection of NETs formation in circulating blood neutrophils of rats 72 h after DAI. **(B)** Proportion of CitH3 + and MPO + double-positive cells in experimental and control groups (*P < 0.05). Grouping: DAI group (*n* = 10) and control group (*n* = 6). CitH3, Citrullinated histone H3; MPO, myeloperoxidase.

Flow cytometry was used to analyze the formation ratio of neutrophil NETs in rat circulating blood (Figures 3A,B). CitH3 + and MPO + were used as markers of NETs formation. The results showed that the average formation ratio of NETs in rat circulating blood was 40% at 72 h after DAI, while the average formation ratio of circulating blood NETs in the control group was 16.7%. The difference was statistically significant (*P* < 0.05).

**FIGURE 4:**
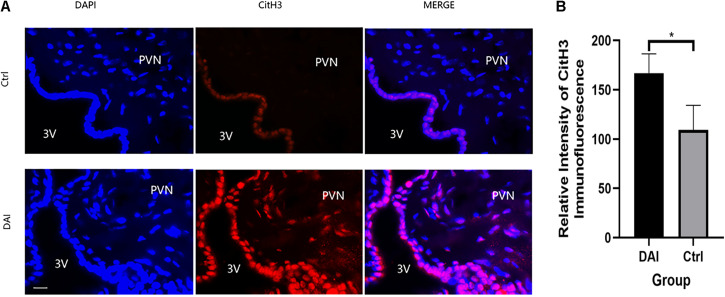
**(A)** Immunofluorescence detection of Citrullinated histone H3 (CitH3) expression in the paraventricular nucleus (PVN) of rats 72 h after DAI; red fluorescent (CitH3) and blue fluorescent, 4′,6-diamidino-2-phenylindole (DAPI) were used to label the tissues in the PVN, 3V (ventriculus tertius) was used to represent the third ventricle of rats; 50 μm scale bar shown. Grouping: DAI group (*n* = 10) and control group (*n* = 6). **(B)** There was a significant difference in CitH3 fluorescence intensity between the DAI and control groups (*P < 0.05).

Immunofluorescence staining of CitH3, a specific protein of NETs in the PVN of rats, showed that there was almost no CitH3 fluorescence in the PVN tissues of the control group, while it increased significantly 72 h after DAI in the PVN of DAI rats (Figure 4).

### Interaction Between LL37 in NETs and Microglial Membrane Receptor P2 × 7

**FIGURE 5:**
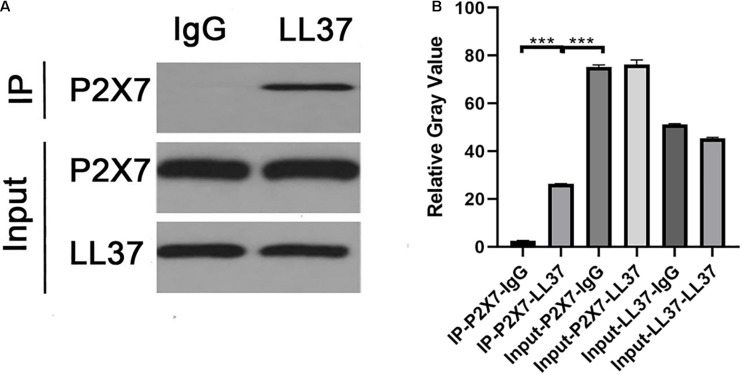
**(A)** The WB results of co-immunoprecipitation of P2 × 7 and LL37. **(B)** The relative gray value of negative control was significantly different from that of P2 × 7- LL37 CO-IP (*p* < 0.05), indicating that there was an interaction between P2 × 7 and LL37 (***P < 0.001).

Co-culture of circulating blood neutrophils with rat glial cells at the optimal time point for observation after DAI showed that there was a correlation effect between P2 × 7 and LL37 in the co-culture system of target cells (Figure 5).

### NETs Can Promote Microglia to Release IL-1β via the Hippo/MST1 Pathway

Inhibition of neutrophil NETs formation by YW3-56 could reduce the expression of MST1 and its downstream effector protein YAP in Hippo/MST1 pathway, and further inhibit the release of IL-1β from microglia. Inhibition of MST1 activation by XMU-MP-1 can decrease the expression of the Hippo/MST1 pathway effector protein YAP in rat neutrophil-microglia co-culture system, and then reduce the IL-1β secretion of microglia.

**FIGURE 6:**
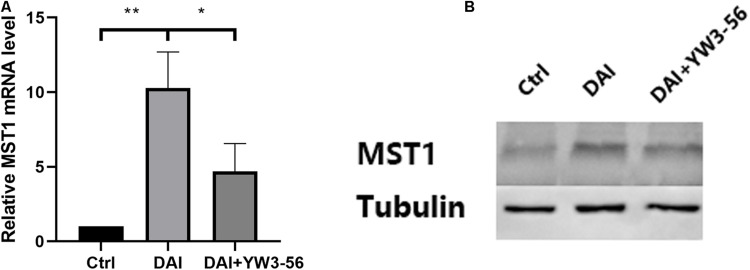
**(A)** The levels of Mammalian STE20-like protein kinases 1 (MST1) mRNA in the control group, DAI group and DAI + YW3-56 group were detected by qPCR. **(B)** The levels of MST1 protein expression in the control group, DAI group and DAI + YW3-56 group were detected by WB (*P < 0.05 and **P < 0.01).

In the co-culture system, DAI rat neutrophils could promote MST1 transcription and expression. When YW3-56 was added to inhibit the formation of NETs, MST1 transcription and expression in the system decreased (Figure 6).

**FIGURE 7:**
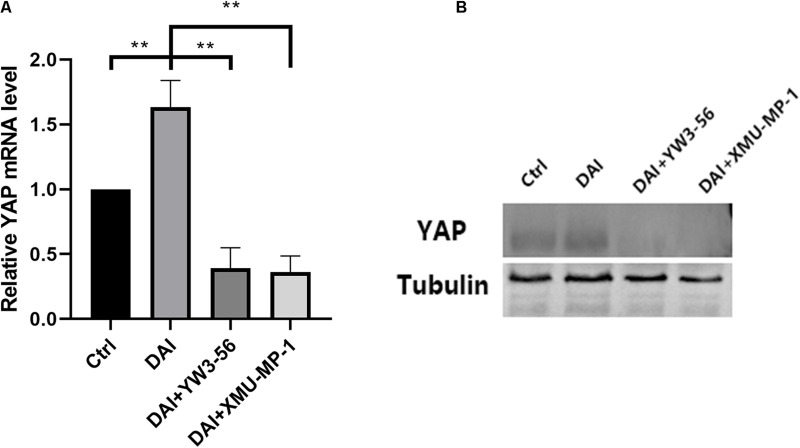
**(A)** Yes-associated protein (YAP) mRNA levels in the control group, DAI group, DAI + YW3-56 group, DAI + XMU-MP-1 group were detected by qPCR. **(B)** YAP protein expression levels in control group, DAI group, DAI + YW3-56 group, DAI + XMU-MP-1 group were detected by WB (**P < 0.01).

In the co-culture system, DAI rat neutrophils could promote the transcription and expression of YAP, which was reduced after adding YW3-56 or XMU-MP-1 (Figure 7).

**FIGURE 8:**
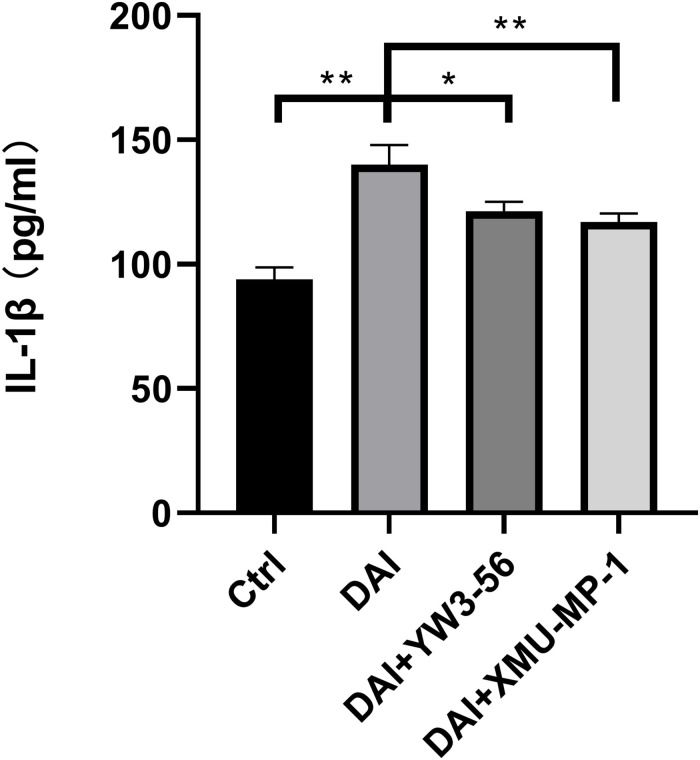
IL-1β expression in cell supernatant of each experimental group (*P < 0.05 and **P < 0.01).

The level of IL-1β in the supernatant of each experimental group was detected by enzyme-linked immunosorbent assay, which was higher in the co-culture system in DAI rats than that in the control group. The level of IL-1β decreased after the inhibition of NETs formation by YW3-56 or XMU-MP-1 (Figure 8).

## Discussion

Sympathetic hyperactivity after TBI is a severe problem in clinical practice. The pathogenesis hypotheses mainly involve diencephalic autonomic epilepsy (Do et al., 2000), cortex-hypothalamus-diencephalon-brainstem disconnection (Meyfroidt et al., 2017), excitatory: inhibitory imbalance (Baguley, 2008), and neuroendocrine (Renner, 2015). These hypotheses are partially reasonable, but they can’t fully explain the phenomenon and lack the exploration of underground pathophysiological mechanisms. Detailed molecular mechanisms are yet unclear. We firstly proved the existence of NETs in the PVN after TBI and examined its effects on the sympathetic hyperactivity. Then, we studied the underlying mechanism of NETs affecting sympathetic excitation. We found that NETs activated microglia through LL37-Hippo/MST1 pathway, promoted the release of IL-1β by microglia and finally contributed to the occurrence of sympathetic excitation. Our previous finding indicates that reactive oxygen species are significantly increased in the PVN after TBI and are critically involved in sympathetic excitation (Chen et al., 2019). We tend to believe that reactive oxygen species in the PVN promote the chemotaxis and formation of NETs, which contributes to sympathetic excitation by the LL37-Hippo/MST1-IL-1β pathway.

The PVN is a key region in the regulation of central nervous system sympathetic excitation in various pathological conditions (Meyfroidt et al., 2017; Koba et al., 2018). The PVN plays an important role in the regulation of the autonomic nervous system and neuroendocrine functions, and it can dispose of the sympathetic activities of its subordinate centers (Meyfroidt et al., 2017; Koba et al., 2018). Levels or functions of excitatory/inhibitory neurotransmitters and their receptors are subtly regulated in the PVN to affect sympathetic nerve activity (Pandit et al., 2015; Busnardo et al., 2016; Zhou et al., 2018). The excitatory neurotransmitter glutamate (Glu) can activate the PVN neurons through NMDA/AMPA receptors and promote sympathetic excitation (Busnardo et al., 2016). Gamma-aminobutyric acid (GABA), on the contrary, can inhibit sympathetic activity through GABA receptors (Pandit et al., 2015). The level of catecholamine in the blood increased slowly from 24 to 48 h after TBI and then increased rapidly from 48 to 72 h. This may be due to the increase of catecholamine caused by stress reaction immediately in the first 24 h after TBI. However, from 48 to 72 h, NETs formed in the PVN, activated microglia, and changed the level and function of neurotransmitters and receptors. This contributed to sympathetic hyperactivity. The concentration of catecholamine increased rapidly because of the sympathetic hyperactivity.

In the animal model of TBI, central neutrophil granulocyte staining was evident in the ventral hypothalamus, periventricular area, meninges, hemispheric commissure, small and large blood vessels in the brainstem, and specific brain structures, including the PVN (Zhang et al., 2013). The high expression of cytokine-induced neutrophil chemoattractant (CINC), a neutrophil-specific chemokine, in the PVN (Jassam et al., 2017), further confirmed that neutrophils can break through the blood-brain barrier and infiltrate the specific areas of the central nervous system in the early stage of injury. Neumann et al. (2014) observed that polymorphonuclear neutrophils (PMNs) were chased and captured by microglia as they entered the brain parenchyma and formed extracellular reticular structures under two-photon fluorescence microscopy. In our experiment, the formation of neutrophil NETs was found in the PVN 24 h after DAI and showed a further increasing trend at 72 h which was the best time point for studying the formation of NETs in the PVN of rats. NETs were detected in circulating blood neutrophils of rats 72 h after DAI. A positive correlation was also found between the concentration of the PVN NETs and blood catecholamine concentrations across time points. A previous study has also found that neutrophil infiltration in the brain parenchyma generally does not occur until 3–5 days after injury (Jassam et al., 2017), which is coherent with our study.

Recent studies have found that NETs can promote CD68 + macrophage activation in adult Still disease (Silva et al., 2018), while microglia are macrophages in the central nervous system (Gosselin et al., 2017). Zhao et al. (2016) also confirmed that Hippo/MST1 signaling pathway can mediate microglial activation after cerebral ischemia-reperfusion injury through IκBα phosphorylation. The Hippo/MST1 signaling pathway is involved in apoptosis, oxidative stress and inflammation. The loss of the MST1 gene in microglia leads to reduction of microglial activation and improvement of neuronal injury after cerebral ischemia-reperfusion injury (Zhao et al., 2016). Similar results have also been confirmed in TBI (Li et al., 2018) and spinal cord injury models in rats (Wang et al., 2017).

LL37 is one of the components of NETs, which can activate astrocytes and microglia and promote the release of pro-inflammatory factors IL-1β and IL-6, chemokines IL-8 and CCL-2 and other substances (Lee et al., 2015). Clinical studies have found that the levels of IL-1β in cerebrospinal fluid and blood begin to rise several hours after TBI and are significantly associated with poor clinical prognosis of patients (Donat et al., 2017). In the co-culture system of rat neutrophils and microglia, LL37 in NETs interacted with microglial membrane receptor P2 × 7, suggesting that LL37 may directly activate microglia through membrane receptor P2 × 7. Inhibition of NETosis by YW3-56 can reduce the expression of MST1, YAP and the release of IL-1β by microglia, indicating that NETs contribute to the activation of microglia and IL-1β secretion in the PVN. A previous study also found that activation of the P2 × 7 purinoceptors in the glial cell is essential for IL-1β release (Neves et al., 2020). Besides, Zhao et al. (2016) found that the Hippo/MSTI signaling pathway can regulate microglial activation. Therefore, it can be speculated that P2 × 7 may mediate the activation of microglia and the release of IL-1β through the Hippo/MSTI signaling pathway. Inhibition of MST1 activation by XMU-MP-1 could inhibit the expression of YAP and the release of IL-1β, suggesting the potential role of the Hippo/MST1 pathway in microglial activation by NETs.

Recent studies have found that IL-1β can promote sympathetic excitation by altering the levels or functions of neurotransmitters such as Glu and GABA and their receptors (Liu et al., 2014; Yan et al., 2019). IL-1β enhances neuronal activity by directly increasing levels of presynaptic Glu release and post-synaptic AMDA receptor expression (Yan et al., 2019). In addition, IL-1β can directly increase the release level of the PVN intracellular Glu and decrease the release level of GABA, thus promoting sympathetic excitation (Liu et al., 2014). Therefore, it is speculated that NETs formed after TBI could activate microglia through LL37-Hippo/MST1 pathway and then prompt IL-1β secretion of microglia. IL-1β regulates the levels or functions of neurotransmitters and their receptors in the PVN, contributing to sympathetic excitation.

Clinically, sympathetic excitation after TBI is characterized by delayed onset and persistence, which generally occurs 48 h to 2 weeks after injury and lasts for up to several months (Lv et al., 2010, 2011a, 2011b; Meyfroidt et al., 2017). In the experiment, we also found that the level of sympathetic excitation and plasma catecholamine increased after DAI. 72 h after injury was the best observation time point for secondary sympathetic excitation. NETs were formed in circulating blood and the PVN after DAI. Notably, Jassam et al. (2017) found that only perivascular and meningeal neutrophil infiltration was present in brain tissue within 24 h after injury, and neutrophil infiltration in brain parenchyma generally did not occur until 3–5 days after injury, which coincided with the time of delayed occurrence of sympathetic excitation.

Although we have tried our best to design a reasonable experimental scheme, there are still some shortcomings. Animals cannot fully reflect various pathophysiological and molecular pathways in the human body. Subsequent clinical trials in humans will bring greater breakthroughs in the pathogenesis of sympathetic excitation after TBI. Moreover, due to the complex composition of NETs, components other than LL37 might also activate microglia to affect the sympathetic excitation after TBI. Although this study indicates that the role of the LL37-P2 × 7/MST1 signaling axis matters, the possible existence of other pathways also deserves further study.

## Conclusion

We suppose that neutrophils infiltrate into the PVN after TBI, thereby inducing neutrophils to release NETs. NETs promote microglial activation and IL-1β release through the LL37-P2 × 7/MST1 pathway, which in turn regulates the level and function of neurotransmitters and enhances the activity of pre-sympathetic neurons, contributing to secondary sympathetic excitation. Further study is essential to enrich and confirm our findings, and more work is necessary before the clinical application.

## Data Availability Statement

The raw data supporting the conclusion of this article will be made available by the authors, without undue reservation.

## Ethics Statement

The animal study was reviewed and approved by the Animal Care and Use Committee of The Second Military Medical University.

## Author Contributions

KZ, YbZ, XH, WC, and XQ preformed the animal surgery. XQ, YlZ, ZL, CW, and JC performed the animal handling, tissue and material preparation, and data collection. KZ, YbZ, XH, LL, and JW performed the data analysis. KZ and YbZ wrote the first draft of the manuscript. All authors contributed to the study conception and design, commented on previous versions of the manuscript, and read and approved the final manuscript.

## Conflict of Interest

The authors declare that the research was conducted in the absence of any commercial or financial relationships that could be construed as a potential conflict of interest.

## Abbreviations

PSH: paroxysmal sympathetic hyperactivity
TBI: traumatic brain injury
NETs: neutrophil extracellular traps
CitH3: Citrullinated histone H3
MST1: Mammalian STE20-like protein kinases 1
DAI: diffuse axonal injury
PVN: paraventricular nucleus
PAD4: Peptidyl arginine deiminase-4
MPO: myeloperoxidase
YAP: Yes associated protein.
